# Accidental displacement of a dental implant into the nasal cavity: Report of a rare case

**DOI:** 10.1002/ccr3.6634

**Published:** 2022-11-23

**Authors:** Yaser Safi, Hamed Mortazavi, Ali Sadeghian, Parham Hazrati

**Affiliations:** ^1^ Department of Oral and Maxillofacial Radiology, School of Dentistry Shahid Beheshti University of Medical Sciences Tehran Iran; ^2^ Department of Oral Medicine, School of Dentistry Shahid Beheshti University of Medical Sciences Tehran Iran; ^3^ School of Dentistry Shahid Beheshti University of Medical Sciences Tehran Iran; ^4^ Dental Research Center, Research Institute of Dental Sciences Shahid Beheshti University of Medical Sciences Tehran Iran

**Keywords:** dental implants, dental implantation, implant migration, nasal cavity

## Abstract

Through radiographic evaluation to discover the location of a displaced implant, it was revealed that the implant had migrated to the middle meatus of the nasal cavity. The patient had no signs or symptoms, and no inflammation was observed radiographically. The implant was removed under endoscopy through the nostril.

## Graphical abstract

Accidental displacement of a dental implant to the maxillary sinus must be treated as soon as possible since it could be moved to the nasal cavity gradually. Additionally, patients affected by this condition might be completely asymptomatic.

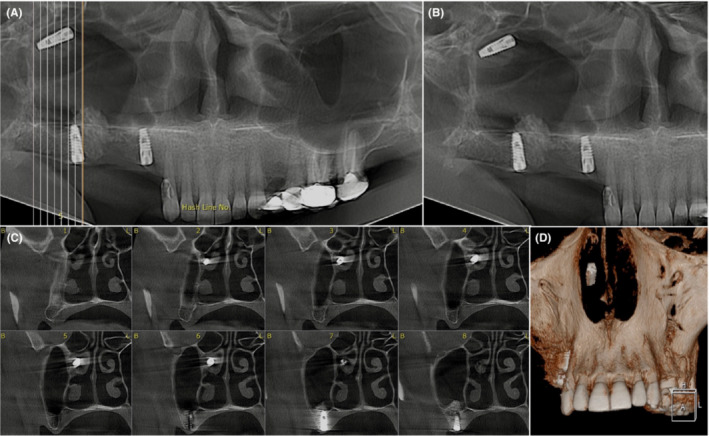

## 1 INTRODUCTION

In dentistry, oral rehabilitation using dental implants has gained more popularity among patients and dentists because of dispelling some drawbacks and limitations of traditional prostheses. Even though clinicians generally consider this treatment to be safe, implants can be accompanied by various problems and complications, such as damaging adjacent neurovascular bundles including inferior alveolar and mental bundles, infection, and migration (i.e., displacement) to abutting anatomical spaces.^1^ Maxillary, ethmoid, and sphenoid sinuses, cranial fossae, orbital floor, and nasal cavity are reported sites of implant migration.^2^, ^3^, ^4^, ^5^, ^6^ There have been many cases of dental implants migrating into the maxillary sinus, while instances of displacement into other craniofacial tissues are sporadic.^5^ Implant migration into the nasal cavity is extremely rare. As far as we know, to this day, only four studies in the literature have reported this unique complication. It has been decades since implants became a frequent routine procedure in everyday dental practice. Regarding this high application rate, only four reported occurrences of this complication indicate the rarity of this condition. Migrated implants could damage vital organs and cause severe or life‐threatening injuries; therefore, it is crucial to identify the exact coordinates of migrated implants to extract them securely and quickly. Because of the complicated anatomy of paranasal sinuses and the nasal cavity, conventional radiographic approaches fail to depict the exact situation, and more advanced imaging techniques are usually required. These imaging modalities necessitate professional interpreting and previous encounter with comparable scenarios in the field.

Here, we report a clinical case of migration of an implant into the nasal cavity, which was discovered when taking three‐dimensional (3D) radiographs to find it after its displacement into the maxillary sinus.

## 2 CASE REPORT

A healthy, 48‐years‐old, non‐smoker, male patient with no related systemic condition was referred to our department by his oral and maxillofacial surgeon in order to take a cone‐beam computed tomography (CBCT) radiograph of an intruded implant into his right maxillary sinus. Six months ago, the patient had undergone open (window) sinus floor elevation surgery at the site of lost teeth 3, 4, and 5 in order to rehabilitate the posterior free‐end edentulous area. The dentist placed three implant fixtures on the corresponding sites 3 months later. The implants of teeth 3 and 5 were successfully placed with proper primary stability, but the implant of region 4 (maxillary second premolar) was displaced into the maxillary sinus because of the dentist's miscue and improper insertion. Afterward, the dentist sutured over the region of the intruded implant, informed the patient, and referred him to an oral and maxillofacial surgeon to remove the implant from his sinus. Whereas the dentist had strongly recommended that the patient should visit the oral and maxillofacial surgeon as soon as possible, the patient recoursed the oral and maxillofacial surgeon with a delay of 3 months and was instructed to take a new CBCT image of the displaced implant. Then, the patient was referred to our oral and maxillofacial radiology department to take a CBCT scan to inform the surgeon about the location of the migrated fixture.

**FIGURE 1:**
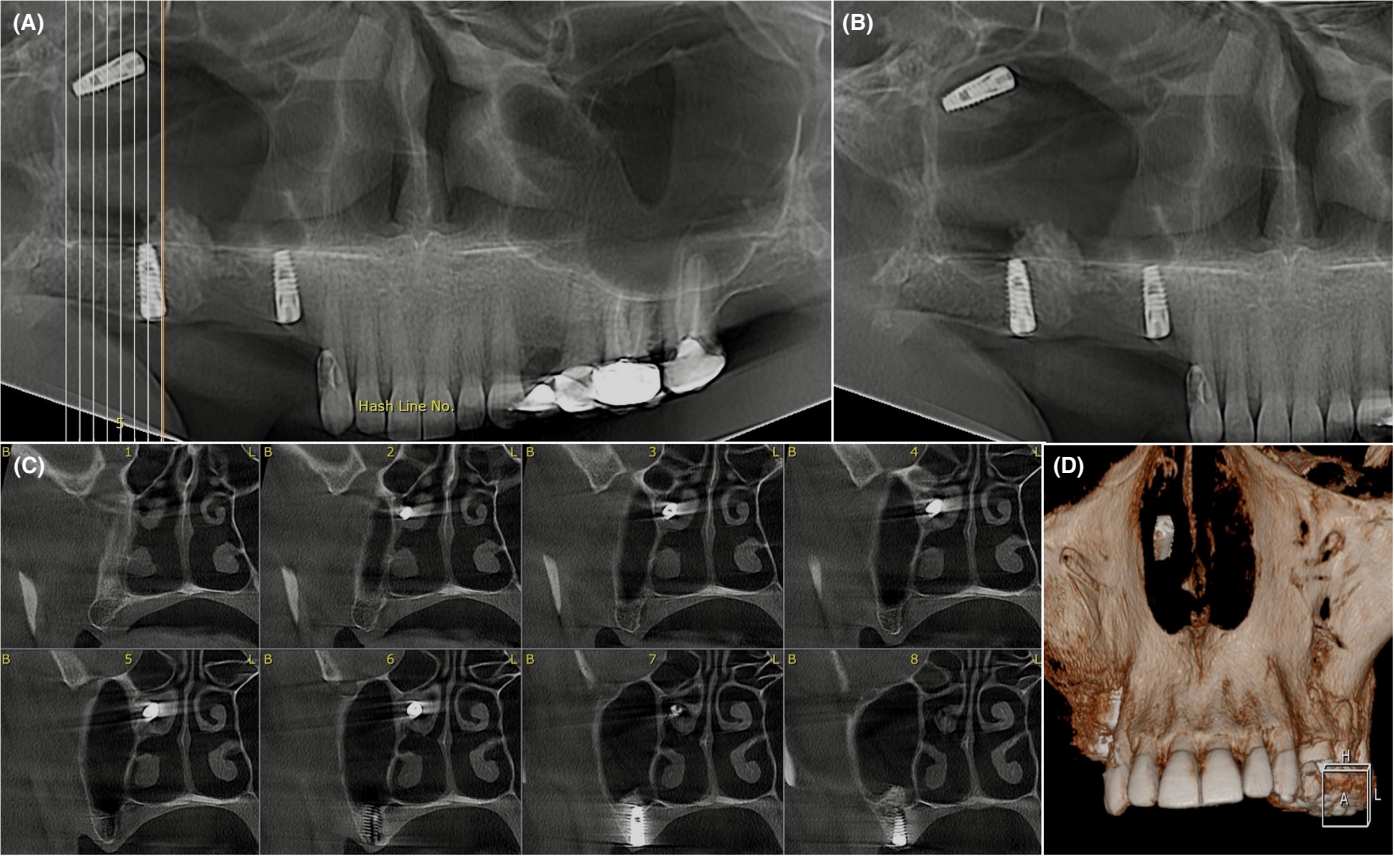
(A) Reconstructed panoramic view from the patient's CBCT radiograph (with a thickness of 25 mm) with Hash lines and (B) without Hash lines and (C) cross‐sectional planes illustrate the migrated implant, which has displaced from the maxillary sinus through the ostium and is currently trapped on the middle concha of the nasal cavity. (D) The position of the implant can be seen in the patient's 3D image.

**FIGURE 2:**
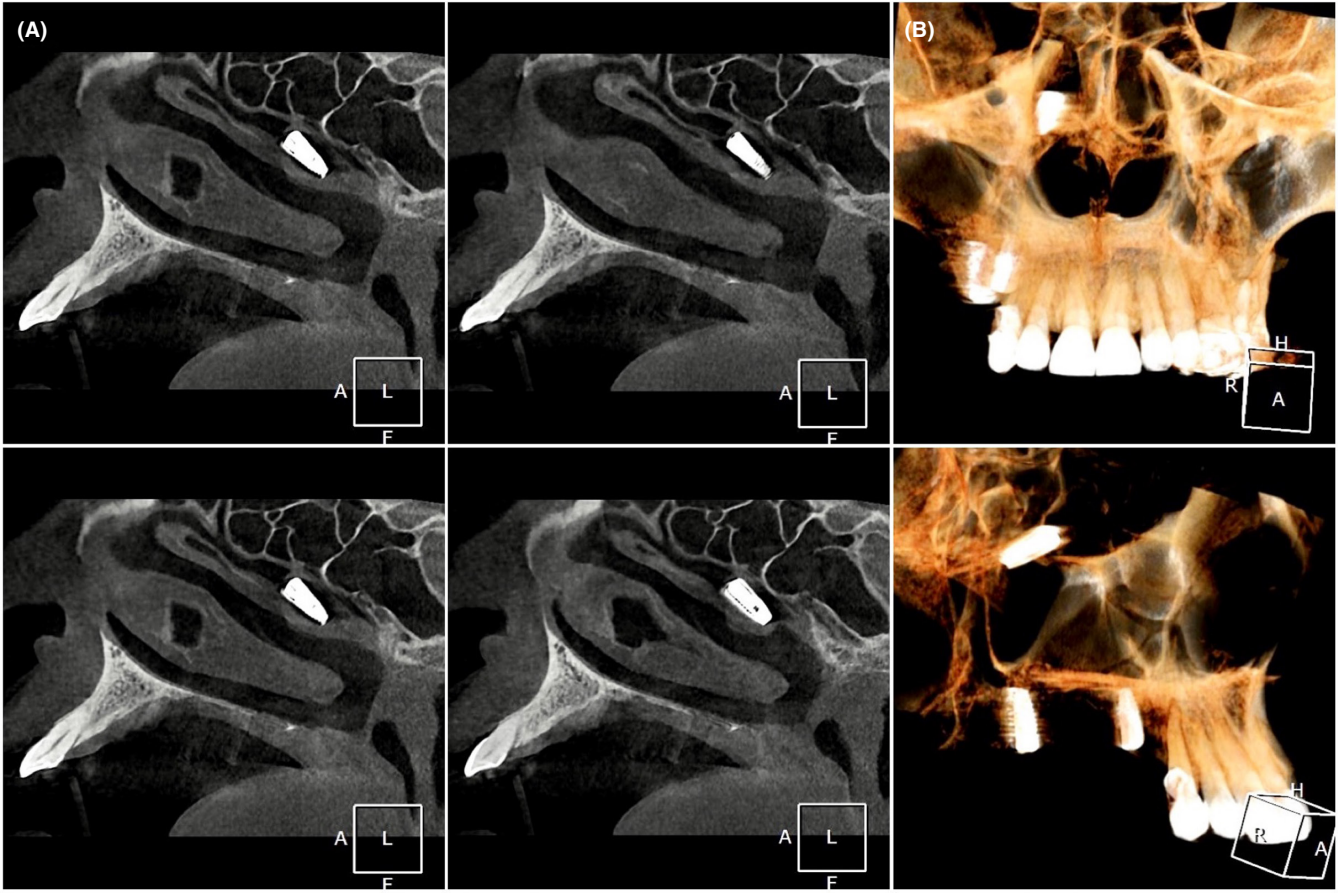
(A) In the sagittal picture series, the trapped migrated implant is witnessed in the right middle concha's region. It is worth mentioning that no inflammatory reaction or mucosal thickening is observed in the mentioned area. (B) In 3D images, the spatial position of the implant is evident.

**FIGURE 3:**
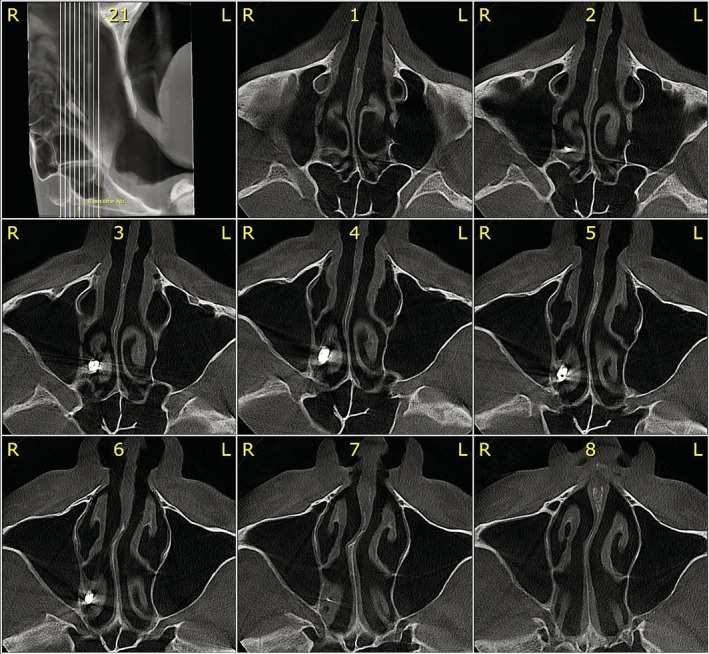
In the axial picture series, position of the migrated implant in the middle meatus is evident. In addition, no inflammatory reaction or mucosal thickening is witnessed.

No extra‐oral or intra‐oral signs or symptoms, such as fever, discomfort, edema, erythema, or suppuration, were observed in the clinical examination. 3D radiographic evaluation revealed that the implant has migrated to the nasal cavity through the ostium of maxillary sinus and is trapped under the middle nasal concha in the middle meatus (Figures 1 and 2). No aberrant opacity or thickening of the sinus lining was observed in the right maxillary sinus (Figure 3). The patient was informed of his situation and was referred to an otorhinolaryngologist for further interventions.

After complete infiltration of the nasal mucosa with local anesthetic solution, the ENT surgeon removed the migrated implant gently under endoscopy by nasal bayonet forceps and Iterson nasal hook through the right nostril. The patient had some nasal bleeding after surgery, but no additional issues occurred. The patient was given an antibiotic therapy of amoxicillin‐clavulanate for 7 days, as well as analgesics and nasal irrigation. Desired healing was observed at the two‐week follow‐up session, and the patient had no discomfort.

Written consent for the publication of radiographic and demographic information of the patient has been obtained.

## 3 DISCUSSION

Implant treatment can have several complications, such as osseointegration failure (loosening), implant fracture, peri‐implantitis, injury to surrounding structures such as nerves and vessels, and aspiration.^1^ One of the less common complications is migration into adjacent structures, most commonly into the maxillary sinus.^1^, ^2^ Direct displacement of implant may occur for various reasons during implant insertion. This might be due to the dentist's lack of skill and experience, inappropriate bone quality, excessive implant tapping, untreated perforation of the sinus lining (Schneiderian membrane), or application of too much force.^4^, ^6^, ^7^, ^8^, ^9^ Cases of implant migration into maxillary sinus are repeatedly reported, but implant displacement into other anatomical spaces like the nasal cavity is scarce.^2^

We reported a case in which the implant was migrated into the maxillary sinus due to inappropriate insertion and a blunder of the dentist. Then, it accidentally moved into the nasal cavity because of the patient's procrastination and attending to the specialist 3 months after his dentist's order.

Up to the present, there are only four reports of implant migration into the nasal cavity in the literature, which indicates how infrequent this complication is, considering the large number of implant treatments done in clinical practice (Table 1). Two of these studies have reported that the implant had migrated into the nasal cavity indirectly from the maxillary sinus through the ostium, as in our case.^3^, ^6^ While in the other two reports, implants have moved directly from the oral cavity to the nasal cavity by perforating the nasal floor.^4^, ^5^

**TABLE 1:** Characteristics of reported cases of dental implant migration to the nasal cavity

Authors	Year	Gender	Age	Insertion site	Migration site	Symptom	Treatment plan	Prognosis
van de Loo et al.^3^	2013	Male	65	Maxillary left incisors and canine area	Indirectly from oral cavity into inferior nasal meatus through maxillary sinus	Fever, pain, and swelling	The patient spontaneously expelled the implant before the operation.	Good
Menezes et al.^5^	2019	Female	37	Maxillary incisive region	Directly from oral cavity into nasal septum	Nasal obstruction, abscess, and pain	Surgical removal of implant+ Antibiotic therapy	Good
Li et al.^6^	2020	Male	23	Right maxilla	Indirectly from oral cavity to common nasal meatus through maxillary sinus	Foul nasal odor	Removal of the implant with endoscopy under local anesthesia+ Antibiotic and anti‐inflammatory therapy	Good
Sanchis & Díaz al.^4^	2021	Female	41	Anterior maxilla	Directly from oral cavity into nasal cavity	Nasal discomfort	The patient spontaneously expelled the migrated implant through the nose	Good
Safi et al.	2022	Male	48	Right maxillary second premolar	Indirectly from oral cavity to middle nasal meatus through maxillary sinus	None	Removal of the implant with endoscopy under local anesthesia+ Antibiotic and anti‐inflammatory therapy	Good

Although implant treatment itself is more prevalent in females, the current literature suggests that the prevalence of this complication is higher in males compared to females; however, this conclusion cannot be considered robust due to the low number of reported cases. It should be noted that the mean age of reported patients of implant migration is approximately one decade lower than the mean age of patients receiving implant treatment.^10^ Among the reported patients who have experienced migration of a dental implant to the nasal cavity, the youngest and oldest are, respectively, aged 23 and 65.

Extraction of the migrated implant by surgical operation was the treatment of choice in all studies, which had good prognosis in every one of these reports^5^, ^6^; nevertheless, in two studies it has been mentioned that the migrated implant is expelled spontaneously.^3^, ^4^ In the study of Sanchis & Díaze, the implant accidentally was thrown off through the nostril,^4^ but in van de Loo et al.'s study, the implant disappeared after being detected in the CBCT.^3^ Most probably, the implant entered the gastrointestinal tract subconsciously and was thus excreted.

Implant migration into paranasal sinuses can cause pain, fungus‐related infections, and sinusitis.^6^ In all reported cases of implant migration into the nasal cavity, patients had symptoms like nasal discomfort, nasal pain, and purulent discharge^3^, ^4^, ^5^, ^6^; on the contrary, the patient presented in this study did not experience any discomfort. The reason for being asymptomatic is uncertain; nonetheless, this could be explained by the short duration of the presence of the implant in the nasal cavity, the absence of sinusitis, and the aseptic surgical placement of the implant.

In this case, a possible reason for implant migration from the maxillary sinus into the nasal cavity could be the ciliary motion of the columnar epithelium of maxillary sinus membrane, which is toward the primary ostium, in combination with the patient's head movements over time, that made the implant move through ostium into the nasal cavity.

Since implant displacement after implant surgery is not uncommon,^4^ it is essential to use appropriate techniques, properly place the implants in a stable and assured situation, and schedule regular follow sessions. In the case of implant migration, 3D imaging modalities, such as CBCT, are necessary to determine the exact position of implant insertion.^5^ Preoperative 3D imaging should be conducted prior to removing the migrated implant to precisely locate the implant. Due to the possibility of additional migration, the period between imaging and surgery should be kept minimum.

It is also vital to extract the migrated implant as soon as possible since it can move into other structures and cause further complications or cause acute and chronic sinusitis.^11^, ^12^ Intraoral surgical and trans‐nasal endoscopic procedures have both been documented for removing implants migrated to the nasal and paranasal cavieties.^13^, ^14^, ^15^

Findings of this report could help dentists and surgeons prevent, diagnose, and manage accidental migration of dental implants into the nasal cavity.

## AUTHOR CONTRIBUTIONS

**Yaser Safi:** Conceptualization; resources; supervision; validation. **hamed mortazavi:** Conceptualization; supervision; writing – review and editing. **Ali Sadeghian:** Writing – original draft; writing – review and editing. **Parham Hazrati:** Project administration; resources; software; supervision; writing – original draft; writing – review and editing.

## CONFLICT OF INTEREST

The authors declare that they have no source of funding for this particular study.

## CONSENT

A written informed consent was obtained from the patient to publish this report in accordance with the journal's patient consent policy.

## Data Availability

All data presented in this study are available from the corresponding author, upon reasonable request.
